# Maternal dexamethasone exposure during pregnancy in rats disrupts gonadotropin-releasing hormone neuronal development in the offspring

**DOI:** 10.1007/s00441-013-1765-9

**Published:** 2013-12-28

**Authors:** Wei Ling Lim, Tomoko Soga, Ishwar S. Parhar

**Affiliations:** Brain Research Institute, School of Medicine and Health Sciences, Monash University Malaysia, Petaling Jaya, 46150 Selangor Malaysia

**Keywords:** Glucocorticoid, GnRH neuron, Dendrite, Preoptic area, Reproduction

## Abstract

The migration of gonadotropin-releasing hormone (GnRH) neurons from the olfactory placode to the preoptic area (POA) from embryonic day 13 is important for successful reproduction during adulthood. Whether maternal glucocorticoid exposure alters GnRH neuronal morphology and number in the offspring is unknown. This study determines the effect of maternal dexamethasone (DEX) exposure on enhanced green fluorescent protein (EGFP) driven by GnRH promoter neurons (TG-GnRH) in transgenic rats dual-labelled with GnRH immunofluorescence (IF-GnRH). The TG-GnRH neurons were examined in intact male and female rats at different postnatal ages, as a marker for GnRH promoter activity. Pregnant females were subcutaneously injected with DEX (0.1 mg/kg) or vehicle daily during gestation days 13–20 to examine the number of GnRH neurons in P0 male offspring. The total number of TG-GnRH neurons and TG-GnRH/IF-GnRH neuronal ratio increased from P0 and P5 stages to P47–52 stages, suggesting temporal regulation of GnRH promoter activity during postnatal development in intact rats. In DEX-treated P0 males, the number of IF-GnRH neurons decreased within the medial septum, organum vasculosom of the lamina terminalis (OVLT) and anterior hypothalamus. The percentage of TG-GnRH neurons with branched dendritic structures decreased in the OVLT of DEX-P0 males. These results suggest that maternal DEX exposure affects the number and dendritic development of early postnatal GnRH neurons in the OVLT/POA, which may lead to altered reproductive functions in adults.

## Introduction

The gonadotropin-releasing hormone (GnRH) neurons located in the preoptic area (POA) are important for the central regulation of fertility in mammals. GnRH peptide released from the hypothalamus stimulates secretion of pituitary gonadotropins, which in turn, promote gonadal hormone secretion and function. GnRH neurons originate from progenitor cells in the olfactory placode during embryonic day 10.5 (E10.5) in mice (Schwanzel-Fukuda and Pfaff 1989; Wray et al. 1989) and E13.5 in rats (Daikoku and Koide 1998). This unique characteristic of embryonic GnRH neuronal migration into the brain is conserved across vertebrates (Parhar 2002). Major development of GnRH neurons occurs between embryonic days E18 and E19 in rats (Jennes 1989), with 500–1,000 neurons observed in the nasal region and forebrain in early stages (Setalo et al. 1992; Tarozzo et al. 1995), reaching approximately 1,300 in the adult brain (Merchenthaler et al. 1984; Wray and Hoffman 1986). GnRH neurons are distributed in a continuum along the medial septum (MS), diagonal band of Broca (DBB), POA to the hypothalamus, with fiber projections extending to the median eminence (ME) and organum vasculosum of the lamina terminalis (OVLT) (King et al. 1982; Witkin et al. 1982). Disrupted embryonic migration and abnormal number or distribution of GnRH neurons in the brain are linked to hypogonadism and reproductive dysfunction (Schwanzel-Fukuda et al. 1989; Wierman et al. 2011).

Identification of a subpopulation of GnRH neurons has helped to understand the functional positioning of GnRH neurons in relation to the regulation of reproduction. Distribution of GnRH neurons across the female rat estrus cycle revealed distinct groupings of these neurons within the hypothalamus during the preovulatory luteinizing hormone (LH) surge (Hiatt et al. 1992). The neuronal activation marker, c-Fos protein, is expressed in a subpopulation of GnRH neurons within the POA and anterior hypothalamus during the steroid-induced LH surge in females (Hoffman et al. 1990) but not in male rats (Hoffman et al. 2005). Furthermore, neurotransmitter γ-amino butyric acid (GABA) and glutamate are involved in the regulation of GnRH neurons. Different GABA_A_ receptor subunits are expressed in the rostral and medial population of GnRH neurons in the POA (Pape et al. 2001) and different subsets of GnRH neurons respond differently to GABA depending on the gender, age, stage of estrus cycle and diurnal rhythm (Sim et al. 2000; Yin et al. 2008; Watanabe et al. 2009). Activation of the *N*-methyl-d-aspartate receptor type 1 (NMDAR1) has been demonstrated in the medial population and not the lateral population of GnRH neurons within the POA (Ottem et al. 2002). These studies highlight that different subpopulations of GnRH neurons within the POA can be differentially regulated, depending on gender and age as well as on different neurotransmitters.

Gestational stress during pregnancy or fetal glucocorticoid exposure adversely affects brain growth and development of the offspring (Seckl 2004; Lupien et al. 2009). Furthermore, gestational stress or fetal glucocorticoid exposure has severe impact on the structural changes of the hypothalamus, including on the sexually dimorphic nucleus of the POA, anterioventral periventricular nucleus and the suprachiasmatic nucleus in male offspring (Reznikov et al. 1999; Rhees et al. 1999; Weinstock 2007). In addition, fetal exposure to glucocorticoid receptor agonist, dexamethasone (DEX) leads to reduced plasma testosterone levels and impaired sexual behavior in male offspring (Lalau et al. 1990; Ward et al. 1994; Holson et al. 1995; Page et al. 2001; Gerardin et al. 2005) as well as delayed pubertal onset in female rodents (Smith and Waddell 2000; Soga et al. 2012). These can be possibly attributed to the consequence of fetal DEX exposure, which delays the development of the hypothalamic–pituitary–gonadal (HPG) axis in the offspring.

DEX is also clinically used to promote fetal lung maturation (Kattner et al. 1992). This, however, has been linked with low birth weight, increased basal activity of the hypothalamus–pituitary–adrenal axis and altered cognitive function during child development (Lupien et al. 2009). Although animal studies have shown long-term consequences of DEX exposure on the adult HPG function and sexual behavior (Seckl 2004; Weinstock 2007; Soga et al. 2012), little is known about the effect of fetal DEX exposure on the development of the GnRH system, which is crucial to understanding the underlying mechanisms of altered adult reproductive functions.

At the level of GnRH neurons, DEX treatment reduces GnRH gene expression, promoter activity and migratory capacity by glucocorticoid receptor (GR) activation in vitro (Chandran et al. 1994; Attardi et al. 1997; Dondi et al. 2005), as well as decreasing GnRH gene expression in vivo (Gore et al. 2006). Glucocorticoids bind to specific domains on the promoter region of target genes that subsequently alter the target gene expression. The distal and proximal negative glucocorticoid response elements, identified within the mouse GnRH promoter, are involved in the GR-mediated suppression of GnRH transcriptional activity (Chandran et al. 1996), suggesting a direct genomic effect of GR within the GnRH neurons. However, it is unknown whether maternal DEX exposure affects GnRH neurons in offspring during the early postnatal stages. Therefore, we hypothesize that maternal DEX exposure may disrupt the GnRH neuronal development in offspring, thereby leading to altered reproductive function and behaviour in adulthood. The present study was therefore designed to determine the effect of maternally administered DEX on GnRH neuronal number, distribution, dendritic changes and promoter activity in male transgenic rat offspring expressing enhanced green fluorescent protein (EGFP) under the control of GnRH promoter.

## Materials and methods

### Animals

Transgenic Wistar rats expressing EGFP under the control of 3.0 kb rat GnRH promoter were used throughout this study. This transgenic colony was established through a generous gift from Masakatsu Kato and Yasuo Sakuma from the University of Tokyo Health Sciences, Japan, in which the GnRH expression in the EGFP cells has been characterized (Fujioka et al. 2003; Kato et al. 2003; Parhar et al. 2005). All rats were housed under a constant temperature of 22 °C and maintained on a 12 h light:12 h dark cycle with access to food and water ad libitum. Adult female rats were time-bred, with presence of spermatozoa in the vaginal smear designated as embryonic day 0 (E0) and day of birth as postnatal day 0 (P0). Sex determination of P0 pups was confirmed by male-specific sex-determining region-Y (*Sry*) gene expression. DNA was extracted from the tail using DNAzol® reagent (Invitrogen, Carlsbad, CA, USA) and PCR was performed using i-PCR Red 5x Master Mix (i-DNA Biotechnology, Singapore) with *Sry* primers (Forward: 5’-CCCGCGGAGAGAGGCACAAGT-3’; Reverse : 5’-TAGGGTCTTCAGTCTCTGCGC-3’) (Hirasawa et al. 1995). The presence of amplification product (146 bp) was detected by 2.0 % gel electrophoresis. All procedures were conducted according to the Guidelines of the Animal Ethics Committee of Monash University (ethics approval number SOBSB/MY/2008/56 and MARP/2011/064).

### Transgene expression

Intact transgenic rats were used to characterize the number and distribution of GnRH immunofluorescence neurons (hereafter referred to as IF-GnRH), EGFP-GnRH neurons (hereafter referred to as TG-GnRH neurons) and GnRH promoter activity expressed as TG-GnRH/IF-GnRH neuronal ratio in the brain across postnatal development. Male and female rats of P0 (*n* = 5/sex), P5 (*n* = 6/sex) and young adult (P47 estrus female, *n* = 3; P52 male, *n* = 3) stages were used. The young adult females were monitored daily for their estrus cycle to determine the estrus phase, while the young adult males were determined by preputial separation as an external sign of pubertal development (Korenbrot et al. 1977).

### Maternal DEX administration

Dexamethasone-21-phosphate disodium salt (DEX; Sigma, St Louis, MO, USA) was suspended in distilled water and administered at a dose of 0.1 mg/kg body weight, which resulted in the impairment of sexual behavior in male offspring (Holson et al. 1995). The pregnant female transgenic rats received subcutaneous injection of DEX or equivalent volume of distilled water for vehicle (VEH) daily from gestation days 13 to 20. P0 male offsprings from the maternally DEX- (DEX-P0, *n* = 7) and VEH-treated (VEH-P0, *n* = 10) groups were used to determine the number and distribution of GnRH neurons in coronal sections of the brain. Additional DEX-P0 (*n* = 2) and VEH-P0 (*n* = 2) males were used to observe the distribution of GnRH neurons along their migratory route from the olfactory region to the POA in sagittal sections.

### GnRH immunofluorescence

GnRH immunofluorescence was performed to confirm the EGFP transgene expression in GnRH neurons throughout this study. The rats were anesthetized with Zoletil–ketamine–xylazine (13.5 mg/kg) and transcardially perfused with 0.1 M phosphate buffer saline (PBS, pH 7.4) containing 1 % heparin, followed by cold 4 % paraformaldehyde (PFA) dissolved in 0.1 M phosphate buffer (PB). The brains were removed, post-fixed in 4 % PFA (72 h for P0, 48 h for P5, 4 h for P47 and P52) and cryoprotected in 30 % sucrose in 0.1 M PB at 4 °C overnight. The brains were then rapidly frozen in powdered dry ice and stored at −80 °C. The brain tissues were serially sectioned in the coronal or sagittal plane (60 μm for P0 and P5; 50 μm for P47 and P52) from the caudal olfactory bulb to the ME from Bregma +4.20 mm to −2.28 mm (Paxinos and Watson 2005) using a cryostat and processed for free-floating immunocytochemistry. The increase in section thickness of P0 and P5 brains, compared to the young adult brains, helped to maintain the rigidity of the sections for the immunostaining procedures and mounting of the sections to the slides. This ensured that all sections from the P0 and P5 rat brains were collected, to minimize error in the counting of GnRH neurons.

The brain sections were incubated in blocking solution (0.2 % Triton X-100 and 2.0 % NGS in PBS) for 1 h, followed by incubation with a polyclonal rabbit antiserum specific for mammalian GnRH (#635.5, diluted 1:3000, gift from Dr. L. Jennes, University of Kentucky, KY, USA) (Jennes 1990) overnight at 4 °C. Sections were then incubated in biotinylated goat anti-rabbit IgG (diluted 1:200; Vectastain ABC Elite kit, Vector Laboratories, Burlingame, CA, USA) for 1 h at room temperature and avidin-biotinylated horseradish peroxidase complex (20 μl/ml) for 45 min at room temperature. The brain sections were further incubated with streptavidin coupled to AlexaFluor 594 (diluted 1:300, Molecular Probes, Eugene, OR, USA) for 30 min and washed with 0.1 M PBS twice, 10 min each. The sections were then serially aligned, mounted onto 3-aminopropylsilane-coated slides (SuperFrost Plus, Fisher Scientific, Pittsburgh, PA, USA) and coverslips were applied with mounting medium (Vectashield, Vector Laboratories, Burlingame, CA, USA).

### GFP immunofluorescence

GFP immunofluorescence was performed to enhance the EGFP transgene signal in the GnRH neurons of P0 and young adult (P52) male transgenic rats (*n* = 1/each) in the coronal brain sections (60 μm for P0; 50 μm for P52). Two sets of the brain sections from P0 and P52 males were processed for GFP immunohistochemistry. The brain sections were washed in 0.1 M PBS, incubated in blocking solution (0.2 % Triton X-100 and 5.0 % NGS in PBS) for 1 h followed by incubation in mouse monoclonal (LGB-1) anti-GFP antiserum (diluted 1:250; ab291, Abcam, Cambridge, MA, USA) for 24 h at 4 °C. Sections were then incubated in biotinylated horse anti-mouse IgG (diluted 1:200; Vectastain ABC Elite kit, Vector Laboratories, Burlingame, CA, USA) for 1 h at room temperature and avidin-biotinylated horseradish peroxidase complex (20 μl/ml) for 45 min at room temperature. The sections were incubated with streptavidin coupled to AlexaFluor 594 (diluted 1:300, Molecular Probes) for 30 min and washed with 0.1 M PBS twice for 10 min each. The sections were then mounted onto 3-aminopropylsilane-coated slides (SuperFrost Plus, Fisher Scientific) and coverslips were applied with mounting medium (Vectashield, Vector Laboratories).

### Antibody characterization

Specificity of GnRH antiserum used in this study has been tested previously (Jennes 1990; Parhar et al. 2005; Soga et al. 2005) by primary antibody preabsorption with mammalian GnRH peptides. Omission of the primary antibody in the initial step resulted in no staining observed in the sections tested. Distribution and cellular morphology of GnRH immunofluorescence in the present study were similar to those of rat GnRH mRNA (Ronnekleiv et al. 1989).

The GFP (clone LGB-1) monoclonal antiserum (directed against purified recombinant GFP antibody) detects the EGFP in formaldehyde-fixed sections and stains a single band of 27 kDA molecular weight on Western blot according to the manufacturer’s technical information. Confocal images of the GFP immunofluorescence (IF-GFP) on young adult TG-GnRH neurons in this study revealed similar cellular distribution as the GFP staining in GFP-GnRH neurons (Cottrell et al. 2006). Omission of the primary antibody in the initial step resulted in no signal observed in the sections tested.

### Cell count of GnRH neurons

The sections collected from the caudal olfactory bulb to the ME were observed under a fluorescence microscope (Nikon Eclipse 90i, Tokyo, Japan) using G-2A and B-2A fluorescence filters to reveal the IF-GnRH neurons (red fluorescence) and TG-GnRH neurons (green fluorescence) respectively. The population of GnRH neurons was identified based on neuroanatomical markers such as the anterior commisure, third ventricle and optic chiasm, across the brain sections to delineate the areas where GnRH neurons are confined. The numbers of IF-GnRH and TG-GnRH neurons were manually counted whereby all GnRH cells cut through the plane of the nucleus were counted in each section. All the GnRH cell counts were further corrected for the overestimation of counts due to the section thickness (50 μm for P47 and P52; 60 μm for P0 and P5) using the Abercrombie’s formula (Guillery 2002). The diameters of GnRH cell bodies were determined for every animal group (P0 male, P0 female, P5 male, P5 female, P52 male, P47 female, VEH-P0 and DEX-P0 males) to calculate the correction factor. Approximately a total of 100 GnRH cells from the MS, OVLT, and AHA region were captured using the confocal microscope (C1si, Nikon, Tokyo, Japan) under 60x water immersion objective lens at 1 μm interval from each group (*n* = 3). The diameters of GnRH cell bodies were then measured from the projected stack of images using Image Pro-Plus v6.0 (Media Cybernetics Incorporation, Bethesda, USA) to obtain the mean cell diameter (± SEM) for each group. Correction factor for cell counts was calculated according to the formula T / T + h, where T = section thickness and h = mean diameter of cell body. Raw cell counts were then multiplied by the correction factor to give the corrected cell count for each group.

The corrected GnRH cell count from serial coronal series was then represented as a histogram to compare the distribution of GnRH neurons in early postnatal and pubertal rat stages (Wray and Hoffman 1986; Gill et al. 2008). The serial coronal sections were aligned using OVLT as a reference point. GnRH cell count from series of three consecutive sections of 60 μm each (P0 and P5) or five sections of 50 μm each (P47and P52) were grouped and plotted as a histogram for the rostral to caudal distribution of GnRH neurons. The cell counts from each section were summed for each animal and averaged across animals for each group to determine the mean ± standard error mean (SEM) values for the IF-GnRH and TG-GnRH neurons. GnRH promoter activity across postnatal development and gender were evaluated using the EGFP expression as a marker expressed as TG-GnRH/IF-GnRH ratio according to Fujioka et al. (2007). For sagittal brain sections, images were captured using a fluorescence microscope (Nikon Eclipse 90i, Tokyo, Japan) and computer software (Adobe Photoshop, Adobe Systems, San Jose, CA, USA) was used to align and combine the images.

### Analysis of dendritic morphology in TG-GnRH neurons

The TG-GnRH neuron dendritic structure was analyzed in VEH-P0 and DEX-P0 males. The sections were viewed with a 40× objective lens of a fluorescence microscope (Nikon Eclipse 90i, Tokyo, Japan) using B-2A fluorescence filters. The TG-GnRH neurons were observed from four serial sections within the OVLT/POA and scored as either soma (no dendrites observed), unipolar, bipolar, or branched dendritic structures following the criteria denoted by Cottrell et al. (2006). The TG-GnRH neuronal dendritic morphology was expressed as a percentage of total TG-GnRH neurons analyzed and averaged across animals for each group to determine the mean (± SEM). The TG-GnRH neurons exhibiting the branch dendritic structure were further confirmed under the high-power imaging of confocal microscopy.

### Confocal imaging

Confocal images of the IF-GnRH and TG-GnRH neurons were acquired using a laser scanning confocal microscope (C1si, Nikon, Tokyo, Japan) with the 488 and 543 nm excitation wavelength respectively. Scans of each wavelength were performed sequentially across optical sectioning to avoid bleed-through between the channels. For the coronal sections, the projected stacks of images of IF-GnRH and TG-GnRH neurons were collected using the 20× objective lens at 2 μm intervals to cover the entire depth of the brain section. For high magnification of GnRH neurons, images of IF-GnRH and TG-GnRH neurons were scanned using the 60× water immersion objective lens, 4× digital zoom function at 0.2 μm intervals to cover the entire depth of the neuron.

Confocal images of the IF-GFP in TG-GnRH neurons were acquired using 60× water immersion objective lens, 4× digital zoom function at 0.2 μm intervals to cover the entire depth of the neuron. Scans of the 488 and 543 nm excitation wavelength were also performed sequentially across optical sectioning to avoid bleed-through between the channels. Brightness and contrast adjustment was made using computer software (Adobe Photoshop, Adobe Systems).

### Statistical analysis

All data are represented as mean ± SEM and statistical analysis was carried out using PASW Statistic 18 (SPSS Inc., Chicago, IL, USA). Statistical significance was determined using two-way analysis of variance (ANOVA) for the total number of IF-GnRH and TG-GnRH neurons as well as the TG-GnRH/IF-GnRH cell ratio, using gender and age as factors, followed by post-hoc Tukey’s test for comparison of the multiple age groups. Two-way ANOVA was also carried out for the distribution of TG-GnRH and IF-GnRH neurons for gender differences in each age group and between VEH-P0 and DEX-P0 males. The significant main effects or interactions from two-way ANOVA were further analyzed using unpaired Student’s *t*-tests. Statistical differences for birth weight, total number of IF-GnRH, TG-GnRH neurons and TG-GnRH/IF-GnRH cell ratio in VEH-P0 and DEX-P0 males were tested using unpaired Student’s *t*-test. Comparison of the number and percentage of different dendritic morphology of TG-GnRH neuronal population between the VEH-P0 and DEX-P0 males was tested using Chi-square test of independence, followed by unpaired Student’s *t*-test for each dendritic morphology. *P* < 0.05 was considered as a significant difference.

## Results

### Transgene expression in the GnRH neurons

**Fig. 1:**
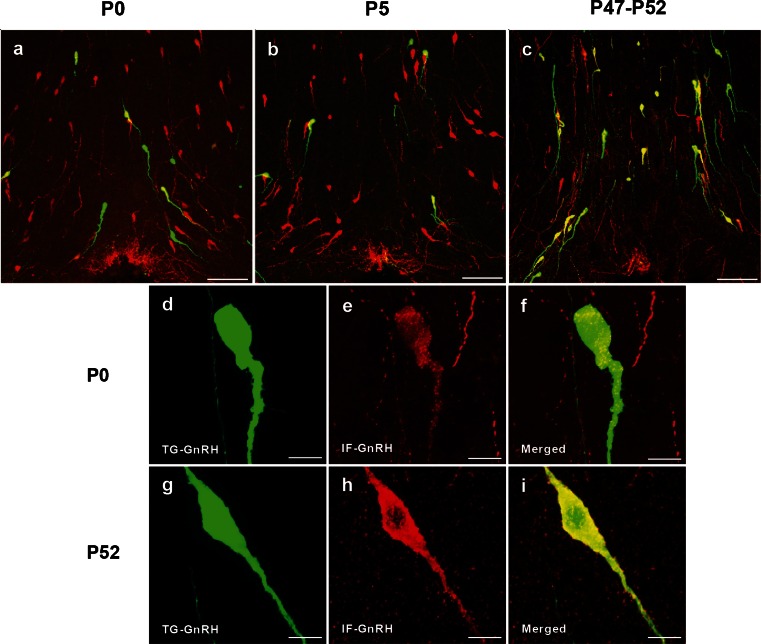
Gonadotropin-releasing hormone immunofluorescence (IF-GnRH) and enhanced green fluorescent protein (EGFP)-GnRH (TG-GnRH) neurons on coronal sections of the preoptic area (POA) in P0, P5 and P52 males. More IF-GnRH neurons (*red*) were observed compared to their respective TG-GnRH cells (*green*) in the POA of P0 (**a**) and P5 (**b**) males. Number of TG-GnRH neurons was comparable to the IF-GnRH neurons in P52 (**c**) male, as most TG-GnRH cells were colocalized with GnRH immunofluorescence (*yellow*) in POA. High-magnification images of TG-GnRH neurons (*green*) double-labeled with IF-GnRH (*red*) and merged images (*yellow*) in P0 (**d**–**f**) and P52 (**g**–**i**) males. Intensity of the IF-GnRH on TG-GnRH neurons in P0 male appeared less compared to the P52 male. *Bars* 100 μm (**a**–**c**) and 10 μm (**d**–**i**)

Double-labeling of the EGFP-positive (green) cells with GnRH immunofluorescence (red) confirmed colocalization of GnRH immunoreactivity in the TG-GnRH cells (yellow) across the postnatal stages (Fig. 1). Few TG-GnRH neurons were observed in the OVLT of P0 (Fig. 1a) and P5 (Fig. 1b) males compared to the P52 males (Fig. 1c). However, a higher number of IF-GnRH neurons was observed compared to the TG-GnRH neurons in the OVLT of P0 (Fig. 1a) and P5 males (Fig. 1b), whereas the number of IF-GnRH neurons was comparable to the TG-GnRH neurons in P52 males (Fig. 1c). The TG-GnRH neurons in P0 and P5 stages were colocalized with the IF-GnRH. High-magnification images of the IF-GnRH colocalization with TG-GnRH neurons in P0 males are shown in Fig. 1d-f, although the intensity of IF-GnRH labeling in TG-GnRH neurons of P0 males appears less compared to that of the P52 males (Fig 1g-i).

**Fig. 2:**
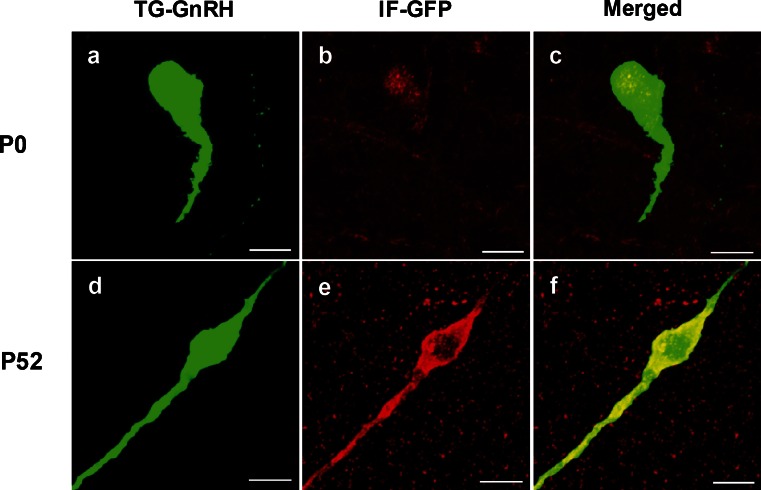
Double labeling of the green fluorescent protein immunofluroscence (IF-GFP, *red*) in the TG-GnRH (*green*) neurons in the POA of P0 (**a**–**c**) and P52 (**d**–**f**) male with the merged images (*yellow*). The IF-GFP staining on TG-GnRH neurons appeared weak and was localized to the nuclear region in P0 male. The IF-GFP staining was distributed across the cytoplasm and along the dendrite of the TG-GnRH neurons in P52 male. *Bars* 10 μm (**a**–**f**)

GFP immunofluorescence was performed to confirm and enhance the EGFP transgene expression in TG-GnRH neurons at different postnatal stages (Fig. 2). Colocalization of IF-GFP (red) in the EGFP-positive (green) cells confirmed the EGFP transgene expression in the TG-GnRH neurons (yellow) in the POA of P0 and P52 males. However, the IF-GFP staining appeared weak and was localized to the nuclear region of the TG-GnRH neurons in P0 males (Fig. 2a-c). Staining of IF-GFP in the TG-GnRH neurons of P52 males was distributed across the cytoplasm and along the dendrite of TG-GnRH neurons (Fig. 2d-f).

### Number of GnRH neurons in postnatal stages of transgenic rats

**Fig. 3:**
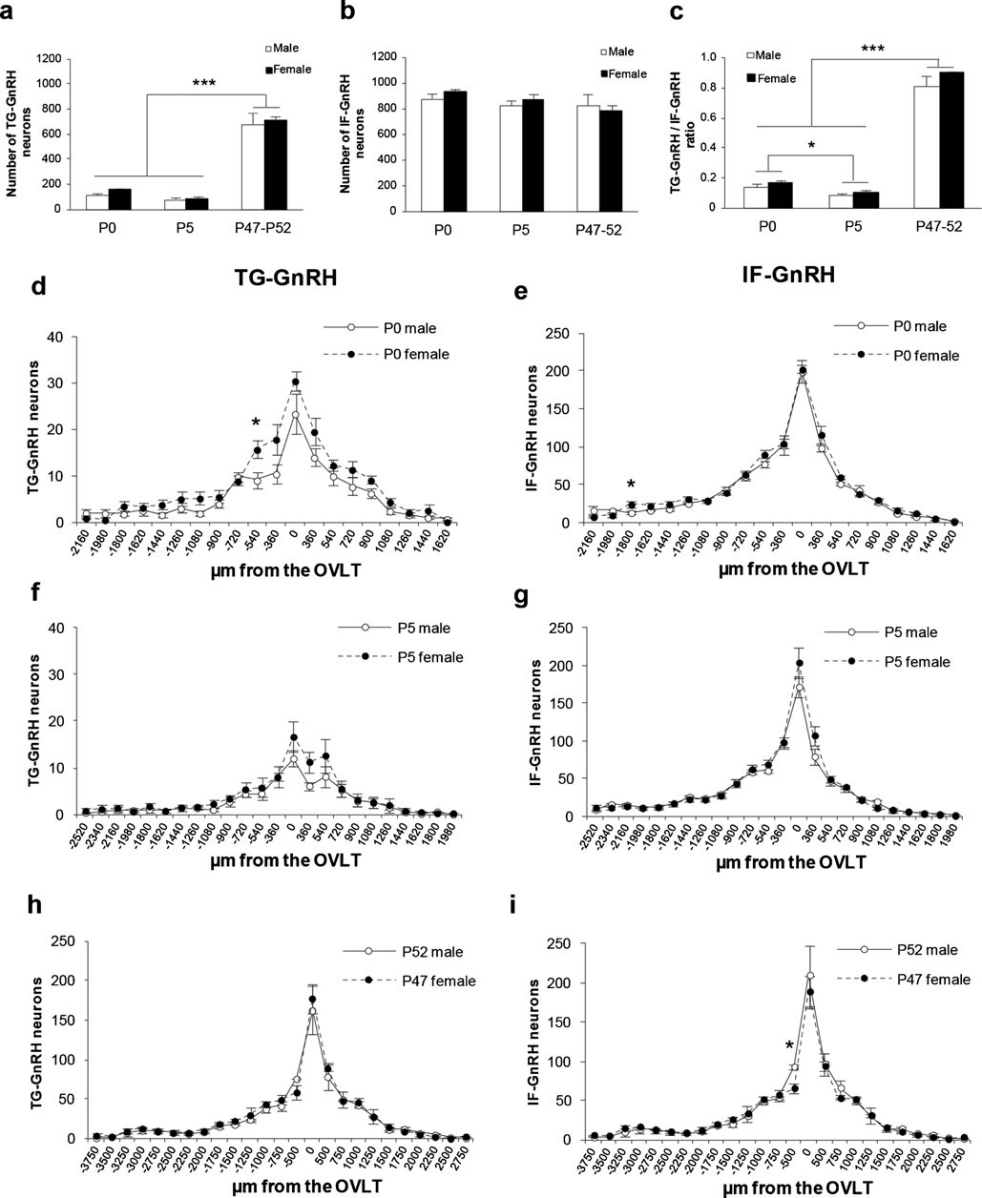
Distribution of the GnRH neurons in P0 (*n* = 5/sex), P5 (*n* = 6/sex) and young adult (P47 female, *n* = 3; P52 male, *n* = 3) transgenic rats. **a** Total TG-GnRH neuronal number was decreased in P0 and P5 stages compared to P47-P52 stage. **b** Number of IF-GnRH neurons did not differ across age and gender. **c** GnRH promoter activity expressed as Tg-GnRH/IF-GnRH neuron ratios was increased in the P42-P52 stage compared to the early P0 and P5 stages. Rostral to caudal distribution of TG-GnRH and IF-GnRH neurons in P0 (**d**–**e**) and P5 (**f**–**g**) male and female, plotted as the number of GnRH neurons in series of 180 μm thickness aligned by organum vasculosom of the lamina terminalis (OVLT). Increased TG-GnRH neuronal number was observed in the rostral region (−540 μm from the OVLT) and IF-GnRH neurons (−1800 μm from the OVLT) in P0 females. **h**–**i** Rostral to caudal distribution of TG-GnRH and IF-GnRH neurons in P47-52 stages, plotted as GnRH neuronal number in series of 250 μm thickness aligned by the OVLT. Gender difference was not observed in the distribution of TG-GnRH and IF-GnRH neurons in P47-P52 stages. Data are represented by the mean ± SEM for each group. *, *P* < 0.05; ***, *P* < 0.001

The total numbers of IF-GnRH and TG-GnRH neurons in the forebrain region of the transgenic rats across the postnatal developmental stages were counted in serial sections collected from the caudal olfactory bulb to the ME. The original raw count of IF-GnRH and TG-GnRH neurons showed similar results to the corrected GnRH cell count across postnatal development in the intact transgenic rat brains. The total number of TG-GnRH neurons varied significantly across postnatal developmental stages [F (2, 22) = 229.61, *P* < 0.001] (Fig. 3a) but no gender difference or gender–age interaction was detected. Tukey post-hoc comparisons revealed significant decrease (*P* < 0.001) in the number of TG-GnRH neurons in the P0 stages (female: 158.9 ± 11.0; male: 115.5 ± 16.7) and P5 (female: 88.8 ± 12.9; male: 67.5 ± 8.2) compared to the young adult stages (P47 female: 710.3 ± 38.6; P52 male: 667.3 ± 94.8). However, there was no difference in the total number of IF-GnRH neurons taken from the same serial sections of transgenic rats across the age group and gender (Fig. 3b). Promoter activity of GnRH neurons across the postnatal developmental stages was expressed as TG-GnRH/IF-GnRH cell ratio (Fig. 3c). The TG-GnRH/IF-GnRH cell ratio varied across postnatal developmental stages [F (2, 22) = 638.30, *P* < 0.001] and gender [F (1, 22) = 8.28, *P* < 0.01] but no gender-age interaction was detected. Tukey post-hoc comparisons revealed a significant decrease (*P* < 0.001) in the TG-GnRH/IF-GnRH cell ratio in the P0 (female: 0.17 ± 0.01; male: 0.14 ± 0.02) and P5 (female: 0.10 ± 0.01; male: 0.08 ± 0.01) compared to the young adult stages (P47 female: 0.91 ± 0.01; P52 male: 0.81 ± 0.06). The TG-GnRH/IF-GnRH cell ratio in the P5 stages was also decreased (*P* < 0.05) compared to the P0 stages.

Distribution of the TG-GnRH and IF-GnRH neurons was examined to observe gender differences across the age group. The distribution of both TG-GnRH and IF-GnRH neurons was seen scattered along the MS, POA and anterior hypothalamus in males and females across the postnatal stages. Two-way ANOVA (location × gender) revealed a significant effect of location [F (19, 154) = 38.46, *P* < 0.001] and gender [F (1, 154) = 23.03, *P* < 0.001] on the distribution of TG-GnRH neurons in P0 males and females (Fig. 3d). The number of TG-GnRH neurons was higher in P0 females in the MS (−540 μm from OVLT, female: 15.7 ± 1.8; male: 9.1 ± 1.8, *P* < 0.05). On the other hand, the distribution of IF-GnRH neurons in P0 males and females (Fig. 3e) revealed a location [F (19, 157) = 197.98. *P* < 0.001] and gender [F (1, 157) = 4.32, *P* < 0.05] difference, as a higher number of GnRH neurons was found in females in the rostral area −1800 μm from OVLT (female: 24.3 ± 4.1; male: 13.6 ± 2.0, *P* < 0.05).

For the P5 stage, two-way ANOVA showed a location [F (23, 229) = 17.25, *P* < 0.001] and gender [F (1, 229) = 6.22, *P* < 0.05] effect on the TG-GnRH neuronal distribution (Fig. 3f). However, a gender difference was not seen in the distribution of IF-GnRH neurons in the P5 stage [location: F (23, 231) = 113.10, *P* < 0.001] (Fig. 3g). Further, two-way ANOVA revealed no gender effect in the distribution of TG-GnRH [location: F (24, 96) = 58.27, *P* < 0.001] (Fig. 3h) and IF-GnRH neurons [location: F (24, 95) = 60.11, *P* < 0.001] (Fig. 3i) in P47 females and P52 males.

### Effect of maternal DEX treatment on GnRH neurons in P0 males

**Fig. 4:**
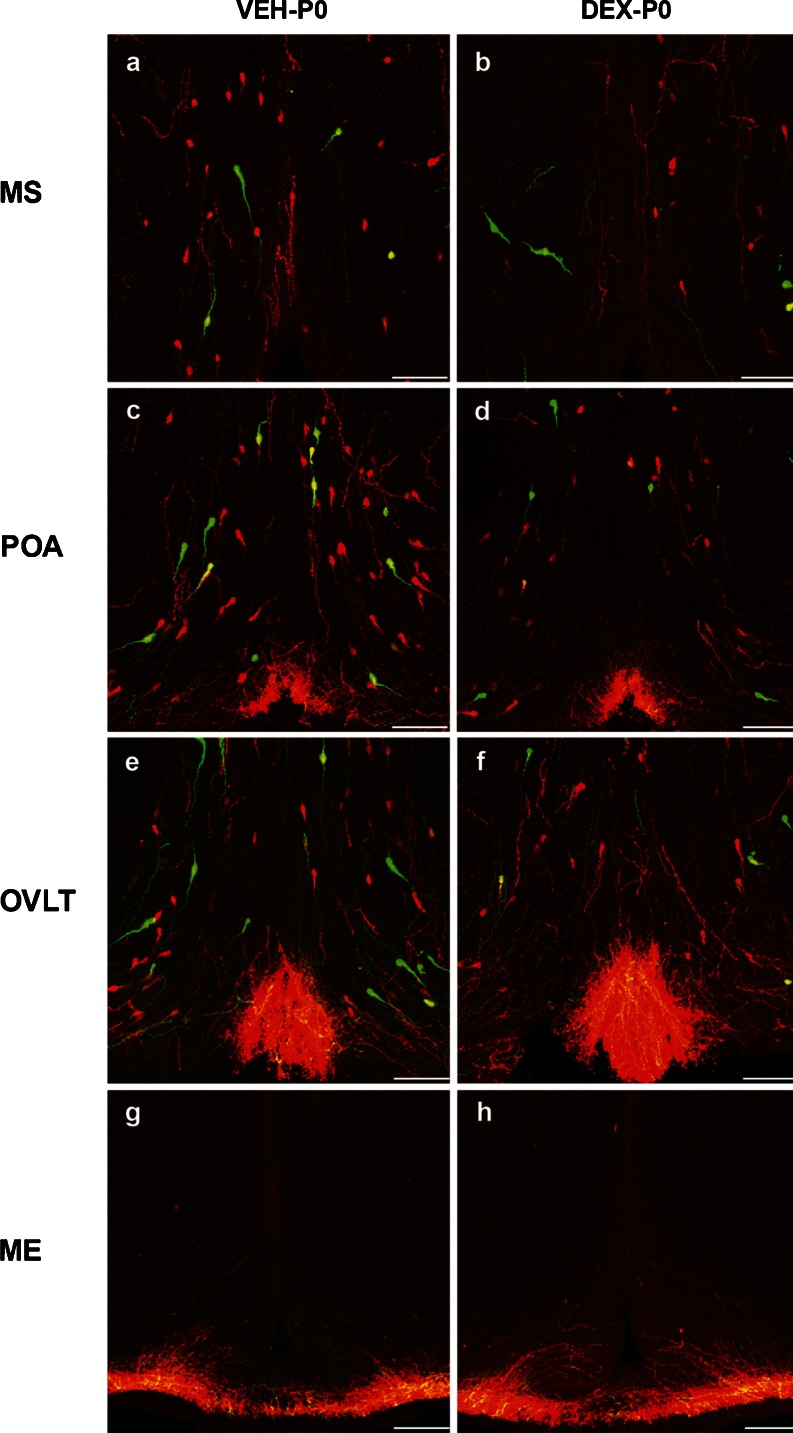
IF-GnRH and TG-GnRH neurons on coronal sections of maternally vehicle- (VEH-P0) and dexamethasone-treated (DEX-P0) P0 male offspring. Number of TG-GnRH neurons (*green*) and IF-GnRH neurons (*red*) observed in medial septum (MS) (**a**–**b**) and POA (**c**–**d**) and OVLT (**e**–**f**) was decreased in DEX-P0 compared to VEH-P0 males. **g**–**h** IF-GnRH fibers projecting to median eminence (ME) were not different in VEH-P0 and DEX-P0 males. *Bars* 100 μm (**a**–**h**)

Maternal DEX exposure resulted in a significant reduction of birth weight in P0 male offspring (VEH-P0: 6.78 ± 0.18 g; DEX-P0: 5.14 ± 0.12 g, *P* < 0.001). Confocal microscopy of coronal sections from the DEX-P0 and VEH-P0 male transgenic rats revealed presence of both TG-GnRH (green) neurons and IF-GnRH (red) neurons in MS (Fig. 4a and b), POA (Fig. 4c and d) and OVLT (Fig. 4e and f). A similar distribution of IF-GnRH neurons was observed in VEH-P0 and DEX-P0 males. Number of IF-GnRH neurons was decreased in the MS and OVLT/POA of DEX-P0 compared to the VEH-P0 males. The distribution of IF-GnRH fibers innervating the ME (Fig. 4g and h) did not differ between VEH-P0 and DEX-P0 males.

**Fig. 5:**
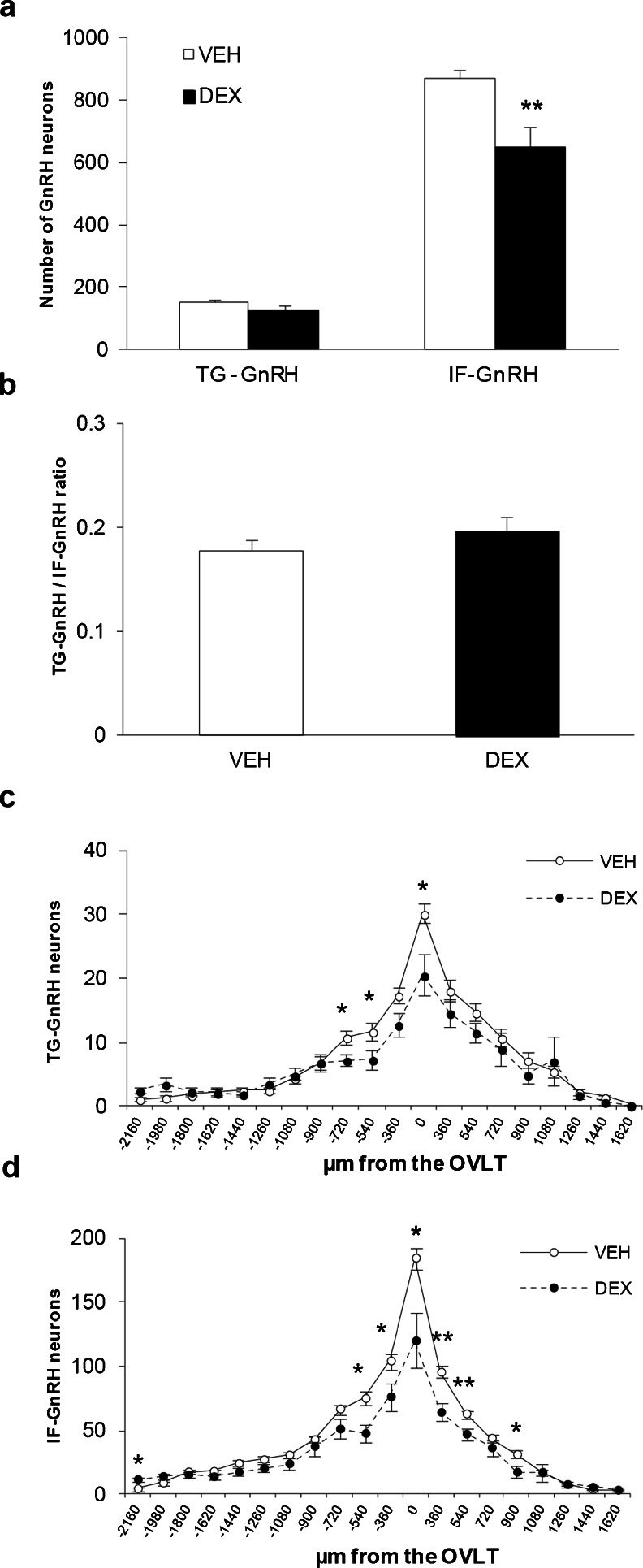
Maternal DEX exposure on number and distribution of IF-GnRH and TG-GnRH neurons in P0 male offspring. **a** Total number of TG-GnRH and IF-GnRH neurons in VEH-P0 (*n* = 10) and DEX-P0 (*n* = 7) males. Maternal DEX exposure decreased the total number of IF-GnRH cells in P0 males but not the TG-GnRH cells. **b** GnRH promoter activity expressed as Tg-GnRH/IF-GnRH neuron ratio did not differ between the VEH-P0 and DEX-P0 males. Rostral to caudal distribution of TG-GnRH (**c**) and IF-GnRH (**d**) neurons in VEH-P0 and DEX-P0 males. The TG-GnRH neurons were decreased in the OVLT and rostral to the OVLT (−540 and −720 μm from OVLT) in DEX-P0 males. Number of IF-GnRH neurons were also decreased in the OVLT, rostral (−360 and −540 μm from the OVLT) and caudal (360 and 540 μm) to the OVLT in DEX-P0 males. Data are represented by the mean ± SEM for each group. *, *P* < 0.05; **, *P* < 0.01 compared to VEH-P0 group

Maternal DEX administration significantly decreased the total number of IF-GnRH neurons in the forebrain of P0 males (VEH-P0: 870.9 ± 25.4; DEX-P0: 648.8 ± 67.0, *P* < 0.01, Fig. 5a) but there was no difference in the total number of TG-GnRH neurons. The effect of maternal DEX exposure was also tested on the promoter activity of GnRH neurons in P0 males, expressed as TG-GnRH/IF-GnRH cell ratio (Fig. 5b) and no difference was observed between the VEH-P0 and DEX-P0 males. The distribution of GnRH neurons in VEH-P0 and DEX-P0 males showed a significant difference at location × treatment interaction for TG-GnRH neurons [treatment: F (1, 292) = 11.48, *P* < 0.01; location: F (19, 292) = 50.35, *P* < 0.001; treatment × location: F (19, 292) = 2.409, *P* < 0.01] (Fig. 5c) and IF-GnRH neurons [treatment: F (1, 295) = 45.40, *P* < 0.001; location: F (19, 295) = 102.48, *P* < 0.001; treatment × location: F (19, 295) = 5.21, *P* < 0.001] (Fig. 5d). A decreased number of TG-GnRH neurons was found in the forebrain of DEX-P0 males (Fig. 5c) within the OVLT/POA (VEH: 30.2 ± 1.5; DEX: 20.5 ± 3.2, *P* < 0.05), MS (−540 μm from OVLT) (VEH: 11.8 ± 1.3; DEX: 7.4 ± 1.6, *P* < 0.05) and in the rostral area −720 μm from OVLT (VEH: 10.9 ± 0.9; DEX: 7.3 ± 1.0, *P* < 0.05). Consistently, the number of IF-GnRH neurons was significantly decreased in the DEX-P0 males (Fig. 5d) within the OVLT/POA (VEH: 184.4 ± 8.6; DEX: 120.2 ± 21.2, *P* < 0.05), rostral area -360 µm from OVLT (VEH: 104.2 ± 6.0; DEX: 76.3 ± 11.1, *P* < 0.05) and MS (−540 μm from OVLT) (VEH: 75.0 ± 5.5; DEX: 47.8 ± 7.2, *P* < 0.05). Decrease in the number of IF-GnRH neurons was also observed in the anterior hypothalamus (360 μm from OVLT) (VEH: 95.5 ± 4.6; DEX: 64.2 ± 6.8, *P* < 0.01), caudal area 540 μm from OVLT (VEH: 63.0 ± 2.9; DEX: 47.2 ± 4.0, *P* < 0.01) and 900 μm from OVLT (VEH: 31.3 ± 3.2; DEX: 17.6.1 ± 4.8, *P* < 0.05) in the DEX-P0 males. A small increase in the IF-GnRH was found in the rostral area −2160 μm from the OVLT (VEH: 4.6 ± 1.5; DEX:11.8 ± 1.8, *P* < 0.05) of DEX-P0 males.

### Effect of maternal DEX treatment on GnRH distribution along their migratory route

**Fig. 6:**
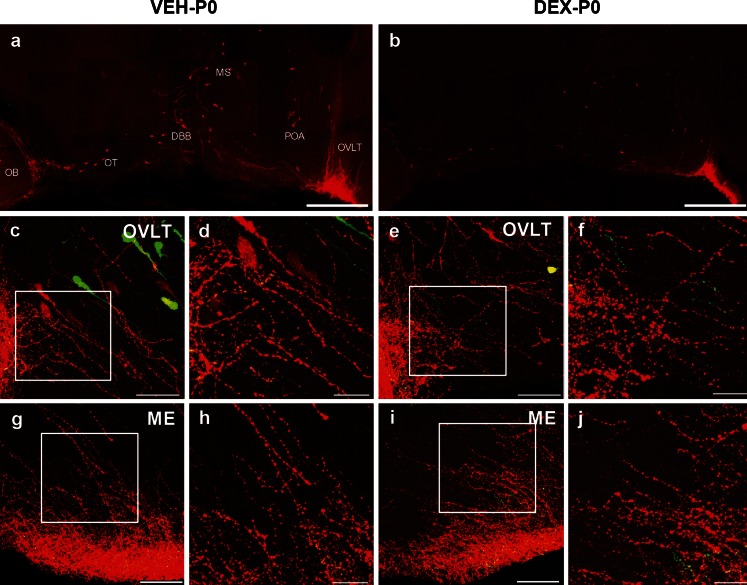
Morphological observations of GnRH neurons and fiber projections in the OVLT and ME in VEH-P0 and DEX-P0 males. Low-magnification photomontage of sagittal sections exhibited IF-GnRH neurons (*red*) along the caudal olfactory bulb (*OB*), olfactory tubercule (*OT*), diagonal band of Broca (*DBB*), MS, and OVLT/POA in VEH-P0 (**a**) and DEX-P0 (**b**) males. Few IF-GnRH neurons were observed along the forebrain distribution in DEX-P0 males. **c**–**j** GnRH fiber projections from coronal sections of OVLT and ME in VEH-P0 and DEX-P0 males. High-magnification of *white insets* illustrating the fiber projections in the OVLT (**d**, **f**) and ME (**h**, **j**) were shown for VEH-P0 and DEX-P0 males respectively. Thicker varicosities of IF-GnRH fibers were observed in lateral OVLT of DEX-P0 (**d**) compared to VEH-P0 (**f**) males. *Bars* 500 μm (**a**-**b**), 50 μm (**c**, **e**, **g**, **i**) and 20 μm (**d**, **f**, **h**, **j**)

IF-GnRH neurons were observed along the migratory route, which included the caudal olfactory bulb, DBB, MS and OVLT/POA in sagittal sections of VEH-P0 and DEX-P0 males (Fig. 6a and b). There was no difference in the distribution but fewer IF-GnRH neurons were found in the DEX-P0 compared to the VEH-P0 males. This observation is comparable with the decreased number of GnRH neurons found in the coronal sections of DEX-P0 males (Fig. 5d). The IF-GnRH fibers were observed within the lateral OVLT (Fig. 6c-f) and ME (Fig. 6g-j) on coronal sections of DEX-P0 and VEH-P0 males. Although the varicosities of IF-GnRH fibers appeared thicker in the lateral part of OVLT of DEX-P0 males (Fig. 6d) compared to VEH-P0 (Fig. 6f), we did not observe any difference in the high-density IF-GnRH fibers within the OVLT region. No differences were observed in the innervations of IF-GnRH fibers in the ME between the VEH-P0 and DEX-P0 males (Fig. 6h and j). Therefore, a quantitative study of the fiber density in the OVLT and the ME region was not carried out.

### Effect of maternal DEX treatment on dendritic structures of GnRH neurons

**Fig. 7:**
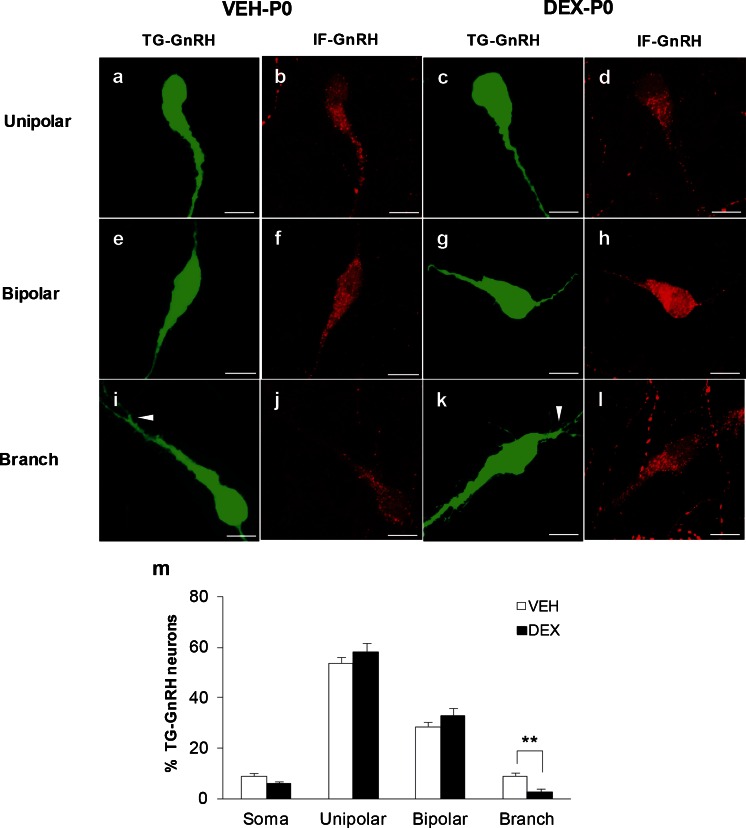
Morphological subtypes of TG-GnRH and IF-GnRH neurons in the OVLT/POA of VEH-P0 and DEX-P0 males. The TG-GnRH neurons were scored as soma, unipolar (**a**, **c**), bipolar (**e**, **g**) or branched dendritic structure (**i**, **k**), with the IF-GnRH (*red*) on the different morphological subtypes of TG-GnRH (*green*) neurons, respectively. *White arrowheads* indicate branching of TG-GnRH neuron dendritic structure. Labeling of IF-GnRH appeared weak in the branched GnRH dendrites, suggesting that the EGFP expression on TG-GnRH neurons is sufficient to visualize the different morphological subtypes. **m** Percentage of TG-GnRH neurons exhibiting branch dendritic structures was decreased in DEX-P0 males. Data are represented by the mean ± SEM for each group. **, *P* < 0.01 compared to VEH-P0 group. *Bars* 10 μm (**a**–**l**)

The GnRH neurons in the P0 stage were observed to exhibit different morphological subtypes such as soma without dendrite, unipolar, bipolar, or branch dendritic structures. The majority of IF-GnRH and TG-GnRH neurons exhibited unipolar and bipolar morphologies (Fig. 7a-h) within the OVLT/POA of P0 males. However, the simple unipolar and bipolar morphology of GnRH cells could be visualized and identified by the IF-GnRH within the OVLT/POA but not to the extent of complex branching along the dendrites of P0 TG-GnRH neurons (Fig. 7i-l). Furthermore, the weak GFP immunofluorescence in the TG-GnRH neurons did not enhance the EGFP signals in GnRH neurons of early P0 stage. Therefore, the EGFP signals in the TG-GnRH neurons were sufficient to permit identification of the different morphological subtypes of GnRH neurons in the VEH-P0 and DEX-P0 males. A chi-square test of independence was performed to examine the relationship between the number of TG-GnRH neurons exhibiting different dendritic morphology and maternal DEX treatment. The relationship between the number of TG-GnRH neurons exhibiting different dendritic morphology and treatment was significant [*X* ^*2*^ (3, *N* = 701) = 16.55, *P* < 0.01]. A significant decrease in the percentage of TG-GnRH neurons exhibiting the branched dendritic morphology was observed in DEX-P0 males (VEH-P0: 8.8 ± 2.8 %; DEX-P0: 2.8 ± 1.4 %, *P* < 0.01, Fig. 7m).

## Discussion

### Number and distribution of GnRH neurons during postnatal stages

The total number and distribution of IF-GnRH neurons in the brain did not differ across gender and postnatal stages in the transgenic rats. It is well-documented that the number of GnRH neurons in the brain is established during birth and maintained throughout adult stages (Wray and Hoffman 1986; Jennes 1989). The brains of the P0 and P5 stages were postfixed for longer periods (48 and 72 hrs) (Ramachandra and Subramanian 2011) to avoid losing brain sections due to tissue softness and to avoid introducing errors to the GnRH counts. Furthermore, our finding that the number and the distribution of IF-GnRH neurons within the MS, POA and the anterior hypothalamus with fiber projections to the OVLT and ME did not change across postnatal stages, P0 to P52, is in agreement with the notion that the GnRH system is established during birth in the EGFP-GnRH transgenic rats.

In the present study, 10–20 % of the total IF-GnRH neurons co-expressed EGFP in both genders of P0 and P5 stage. The percentage of IF-GnRH neurons expressing EGFP increased to approximately 80 % of the IF-GnRH population in both genders of the P47/P52 stage, which is consistent with previous studies using the same line of transgenic rats (Fujioka et al. 2003; Parhar et al. 2005). Fewer numbers (25-35 %) of EGFP-expressing GnRH neurons observed in the fetal stage of these transgenic rats (Fujioka et al. 2007) suggest lower GnRH gene transcriptional activity during the early stage compared to the adult stage. The EGFP expression in GnRH neurons was not confined to a specific distribution pattern or anatomical position within the brain in different age groups. Further, the presence of subpopulations of IF-GnRH neurons expressing EGFP suggests heterogeneity within the GnRH population.

The number of IF-GnRH neurons did not change across gender and age but the number of TG-GnRH neurons increased from the P0 and P5 stages to the young adult stages, which suggests an increase in EGFP expression in GnRH neurons. The TG-GnRH/IF-GnRH cell ratio significantly changed across the postnatal age groups, suggesting increased GnRH promoter activity during postnatal development. Thus, the EGFP-positive/GnRH-positive cell ratio is a reliable marker of GnRH promoter activity (Fujioka et al. 2007). Active GnRH promoter activity and gene transcription are also seen to increase across developmental stages in intact male and female rats (Gore et al. 1999). Furthermore, transgenic mice with human GnRH promoter fused to the luciferase reporter gene show robust increase in luciferase expression prior to pubertal maturation (Wolfe et al. 1996).

Since all GnRH neurons do not express EGFP, it is possible that the 3.0 kb GnRH promoter construct is insufficient to drive EGFP expression in the entire GnRH population. It is possible that the current GnRH promoter lacks the two upstream enhancers described at the transcription start site of the GnRH gene (Iyer et al. 2010). Furthermore, we speculate that the activation of the GnRH gene transcription using different promoter segments could give rise to subpopulations of GnRH neurons expressing EGFP (Parhar et al. 2005). GnRH promoter activity is regulated by transcription factors during the embryonic and postnatal stages (Kelley et al. 2000; Lee et al. 2001; Givens et al. 2005); therefore, it is possible that the interactions between transcription factors and upstream enhancers contribute to the subpopulation specific expression of GnRH in the brain (Iyer et al. 2010).

### Maternal DEX exposure on positioning of GnRH neurons

Maternal DEX administration decreased the number of IF-GnRH and TG-GnRH neurons within the MS, OVLT and anterior hypothalamus of P0 male offspring. The glucocorticoid receptor (GR) is expressed in 10–20 % of GnRH neurons in vivo (Ahima and Harlan 1992; Dondi et al. 2005); therefore, the decrease in the number of IF-GnRH neurons by approximately 20 % in DEX-P0 males suggests that the maternal DEX exposure could have an indirect or a direct effect on GnRH neurons through GR. DEX exposure decreases migration of immortalized GnRH neurons through remodeling of actin fibers and cytoskeletal proteins (Dondi et al. 2005). The decrease in the number of GnRH cells in the P0 male brain could be due to the altered migratory route of GnRH neurons by DEX exposure during embryonic days E13–E20, which coincides with the period of GnRH neuronal migration in rats (Yoshida et al. 1995). Migratory deficit of embryonic GnRH neurons is demonstrated by the decrease in GnRH neuronal population in the adult brain, which partially affects sexual organ maturation, pubertal onset and fertility (Tsai et al. 2005; Pierce et al. 2008).

In the sagittal brain sections of P0 males, no aberrant accumulation of GnRH neurons was observed in the rostral region of the DEX-P0 brain, as would be expected if migratory deficits were to occur due to DEX exposure. We did not harvest the nasal area of VEH-P0 and DEX-P0 males to examine the possible presence of GnRH neurons in this region. As DEX was administered during the late embryonic stage, which coincides with the period of GnRH migration, there is a possibility that GnRH neurons that failed to reach their target area within a restricted time frame could have been eliminated by cell death.

Indeed, prenatal stress induces neuronal apoptosis in the paraventricular nucleus of the hypothalamus and alters axonal morphology in the ME of fetal rats (Fujioka et al. 1999). Therefore, the decrease in the number of GnRH neurons in the OVLT/POA of DEX-P0 males can be attributed to apoptosis induced by glucocorticoid. Furthermore, prenatal stress abolishes testosterone surge in male fetuses (Ward and Weisz 1980), thereby possibly reducing both testosterone and estradiol levels in the brain. This could lead to cell death of GnRH neurons within the OVLT/POA, suggesting an indirect effect of maternal DEX exposure on GnRH neurons. Further studies are therefore needed to understand how glucocorticoid exposure in utero affects the final GnRH numbers and distribution, either by disrupting the migratory process and/or altering the survival of GnRH neurons.

Early-life stress and DEX exposure impairs sexual performance in male and female rat offspring, which includes a decrease in the number of intromissions and ejaculations (Ward et al. 1994; Holson et al. 1995; Gerardin et al. 2005) as well as irregular estrus cycle and delayed pubertal onset (Herrenkohl 1979; Smith and Waddell 2000; Iwasa et al. 2011; Soga et al. 2012). The decreased number of IF-GnRH neurons in the DEX-treated P0 male offsprings further supports the decreased hypothalamic GnRH levels and pituitary LH secretion in fetal and adult male offsprings (Lalau et al. 1990; Page et al. 2001; Shono and Suita 2003), which result in fertility decline during adulthood. Therefore, we would anticipate changes in the LH and FSH cells of male offsprings exposed to prenatal DEX during development. However, no change was detected in the hypothalamic *Kiss1*, *Kiss1r* and *GnRH* mRNA levels in female rat offsprings exposed to maternal DEX treatment (Iwasa et al. 2011). This discrepancy with the present findings is probably due to the route of DEX administration. Iwasa and coworkers (2011) administrated DEX to pregnant rats via drinking water, which probably results in a low DEX dosage exposed to the fetus as opposed to subcutaneous injections to pregnant females. Maternal DEX exposure has different effects on male and female offspring, which suggests possible gender difference in the HPG axis. Indeed, neonatal DEX administration delays pubertal onset in female mice via decreased GnRH levels, mediated by increased GnIH cell number and receptor expression in the POA, suggesting different underlying mechanisms of early-life DEX exposure on the development of the HPG axis (Soga et al. 2012).

The total number of TG-GnRH neurons along with the TG-GnRH/IF-GnRH cell ratio did not differ between the VEH-P0 and DEX-P0 males, suggesting that maternal DEX exposure does not affect the GnRH promoter activity in the early P0 stage. DEX treatment alters post-transcriptional GnRH processing in GT1-1 cells in vitro and reduces GnRH mRNA transcripts (Park et al. 2009). In contrast, DEX administration does not alter GnRH mRNA expression in the GN11 cell (model of immature, highly migratory GnRH neuron), while DEX decreases GnRH mRNA expression in the GT1-7 cell (model of mature, terminally differentiated GnRH neuron), indicating that glucocorticoids possibly have different effects depending on the developmental stages of GnRH neurons (Dondi et al. 2005). This discrepancy of DEX effect on the GnRH promoter activity could be due to the different developmental stages of GnRH neurons used in these studies.

The lack of effect of maternal DEX treatment on the total number of TG-GnRH suggests that the rat GnRH promoter tagged to EGFP reporter does not contain a functional glucocorticoid response element (GRE) to mediate the DEX effect. Indeed, the GRE consensus sequences (Alheim et al. 2003) were not detected in the rat GnRH promoter construct (nucleotide −3032 to +116) tagged to the EGFP reporter (Kepa et al. 1992) using the basic local alignment search tool (BLAST). Two negative GREs (nGREs) have been identified in the mouse GnRH promoter that contribute to the glucocorticoid-mediated repression but do not show homology to the known GRE sequences (Chandran et al. 1996). Additionally, the glucocorticoid receptor does not bind directly to the GREs but mediates its repressive effect in association with other multi-protein complexes in the nGREs (Chandran et al. 1996). This suggests that the GC-mediated effect on GnRH promoter is determined by multiple factors, in which the length of the rat GnRH promoter tagged to EGFP, is probably insufficient to mediate the repression of GnRH promoter and thereby alter the number of TG-GnRH neurons in P0 males.

### Maternal DEX exposure on dendritic branching of GnRH neurons

Despite the loss of GnRH neurons within the OVLT/POA region, GnRH immunoreactive fiber projections to the ME did not differ between DEX-P0 and VEH-P0 males, suggesting that the release of GnRH peptide is not affected by maternal DEX treatment. The density of IF-GnRH fibers within the OVLT region was not altered by maternal DEX treatment; however, thicker varicosities of the IF-GnRH fibers were observed within the lateral OVLT of DEX-P0 males, possibly due to the suppression of the GnRH peptide released from the OVLT (Rotsztejn et al. 1977).

The percentage of TG-GnRH cells with branched dendrite structures in the OVLT/POA was less in the DEX-P0 males, suggesting that dendritic branching and development of GnRH neurons is probably disrupted by maternal DEX treatment in the early P0 stage. The number of unipolar and branched GnRH neurons in the POA changes with developing age. Long dendrite projections accompanied by increased number of spines indicate that postnatal dendritic remodeling and increased excitatory inputs to GnRH neurons are important for pubertal development (Cottrell et al. 2006). Therefore, in prenatally DEX-treated P0 males, a decrease in the number of GnRH neurons exhibiting branched dendritic structures possibly alters the excitatory inputs to the GnRH neurons, which potentially affects the establishment of the GnRH neuronal network and inputs regulating the GnRH function during development.

In summary, maternal DEX exposure during the late gestational stages decreased the number of GnRH neurons within the MS, OVLT/POA and anterior hypothalamus of P0 males. In addition, there were fewer GnRH neurons with branched dendritic structures within the OVLT/POA of DEX-P0 males. Therefore, excess glucocorticoid exposure during late gestational stages affects the total number and dendritic development of GnRH neurons in postnatal males, which possibly contributes to the altered reproductive function and behavior during adulthood.
